# Predictive network modeling in human induced pluripotent stem cells identifies key driver genes for insulin responsiveness

**DOI:** 10.1371/journal.pcbi.1008491

**Published:** 2020-12-23

**Authors:** Ivan Carcamo-Orive, Marc Y. R. Henrion, Kuixi Zhu, Noam D. Beckmann, Paige Cundiff, Sara Moein, Zenan Zhang, Melissa Alamprese, Sunita L. D’Souza, Martin Wabitsch, Eric E. Schadt, Thomas Quertermous, Joshua W. Knowles, Rui Chang

**Affiliations:** 1 Stanford University School of Medicine, Division of Cardiovascular Medicine, Cardiovascular Institute, and Diabetes Research Center, Stanford, California, United States of America; 2 Department of Genetics and Genomic Sciences, Icahn School of Medicine at Mount Sinai, New York, New York, United States of America; 3 Liverpool School of Tropical Medicine, Pembroke Place, Liverpool, United Kingdom; 4 Malawi—Liverpool—Wellcome Trust Clinical Research Programme, Blantyre, Malawi; 5 Department of Neurology, University of Arizona, Tucson, Arizona, United States of America; 6 The Center for Innovations in Brain Sciences, University of Arizona, Tucson, Arizona, United States of America; 7 Vertex Pharmaceuticals, Boston, Massachusetts, United States of America; 8 Department of Cellular, Developmental and Regenerative Biology, Black Family Stem Cell Institute, Icahn School of Medicine at Mount Sinai, New York, New York, United States of America; 9 Department of Pediatrics and Adolescent Medicine, Division of Pediatric Endocrinology, Ulm University, Ulm, Germany; 10 Institute of Genomics and Multiscale Biology, Icahn School of Medicine at Mount Sinai, New York, New York, United States of America; 11 INTelico Therapeutics LLC, Tucson, Arizona, United States of America; University of Michigan, UNITED STATES

## Abstract

Insulin resistance (IR) precedes the development of type 2 diabetes (T2D) and increases cardiovascular disease risk. Although genome wide association studies (GWAS) have uncovered new loci associated with T2D, their contribution to explain the mechanisms leading to decreased insulin sensitivity has been very limited. Thus, new approaches are necessary to explore the genetic architecture of insulin resistance. To that end, we generated an iPSC library across the spectrum of insulin sensitivity in humans. RNA-seq based analysis of 310 induced pluripotent stem cell (iPSC) clones derived from 100 individuals allowed us to identify differentially expressed genes between insulin resistant and sensitive iPSC lines. Analysis of the co-expression architecture uncovered several insulin sensitivity-relevant gene sub-networks, and predictive network modeling identified a set of key driver genes that regulate these co-expression modules. Functional validation in human adipocytes and skeletal muscle cells (SKMCs) confirmed the relevance of the key driver candidate genes for insulin responsiveness.

## Author summary

Insulin resistance is characterized by a defective response (“resistance”) to normal insulin concentrations to uptake the glucose present in the blood, and is the underlying condition that leads to type 2 diabetes (T2D) and increases the risk of cardiovascular disease. It is estimated that 25–33% of the US population are insulin resistant enough to be at risk of serious clinical consequences. For more than a decade, large population studies have tried to discover the genes that participate in the development of insulin resistance, but without much success. It is now increasingly clear that the complex genetic nature of insulin resistance requires novel approaches centered in patient specific cellular models. To fill this gap, we have generated an induced pluripotent stem cell (iPSC) library from individuals with accurate measurements of insulin sensitivity, and performed gene expression and key driver analyses. Our work demonstrates that iPSCs can be used as a revolutionary technology to model insulin resistance and to discover key genetic drivers. Moreover, they can develop our basic knowledge of the disease, and are ultimately expected to increase the therapeutic targets to treat insulin resistance and type 2 diabetes.

## Introduction

Insulin resistance is necessary for the development of the metabolic syndrome and type 2 diabetes (T2D), and is a major risk factor for cardiovascular disease [1], which together represent a modern pandemic. While genome-wide association studies (GWAS) have identified a large number of genomic loci associated with T2D-related traits, most of these signals are associated with pancreatic β-cell function and insulin secretion rather than with insulin resistance [2]. While a few insulin resistance genes have been identified [3–6], the underlying genetic architecture of insulin resistance remains unknown.

To fill this gap, we sought to take advantage of a large library of induced pluripotent stem cells (iPSCs) derived from individuals across the spectrum of insulin sensitivity who have also undergone GWAS genotyping [7,8]. We have fully characterized these iPSC lines and demonstrated determinants of iPSC transcriptional variability. For instance, we found that the highest across individual contribution to variability in our cohort was enriched for metabolic functions [9].

These results prompted us to more specifically analyze the gene expression patterns and networks associated with the insulin sensitivity status of the iPSC donors. For complex conditions like insulin resistance with polygenic susceptibility, systems biology and network modeling, integrating multiscale-omics data like genetic and transcriptomic data, provide a useful context in which to interpret associations between genes and functional variation or disease states [9–13]. Therefore, the reconstruction of molecular networks can lead to a more systematic and data driven characterization of pathways underlying disease, and consequently, a more comprehensive approach to identifying and prioritizing therapeutic targets [12,13]. Recent advances in co-expression and causal/predictive network modeling [9,11,12,14] allow us to take such an approach. The work described here links complex disease phenotypes from highly characterized subjects to concomitant molecular networks that can then be used to uncover coherent, functional molecular sub-networks and their key driver genes that ultimately determine the clinical phenotypes.

**Fig 1:**
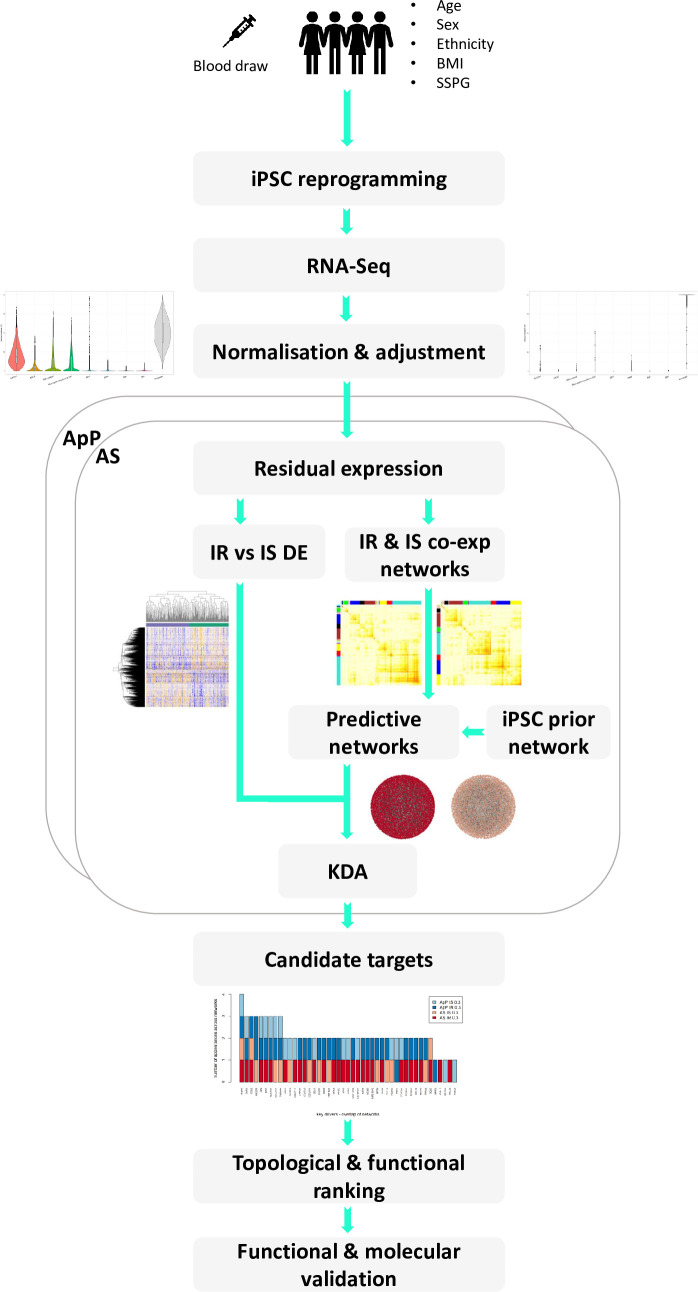
Flowchart detailing the individual steps of the integrative predictive network modeling analysis pipeline and functional molecular validation: sample collection, data generation, data normalization, differential expression analysis, co-expression networks, predictive networks and key driver analysis, prioritization of KDs and molecular and functional validation.

In summary, we performed differential expression analyses between insulin resistant (IR) and insulin sensitive (IS) iPSCs, built co-expression networks to systematically organize the data into coherent modules enriched for insulin sensitivity associated functions and implemented key driver analyses to identify genes that control and regulate critical aspects of the IR and IS networks. Finally, we empirically validated the constructed networks in iPSCs and the prioritized key drivers through insulin responsiveness associated functional assays in human adipocytes and skeletal muscle cells (SKMCs)**(Fig 1)**.

## Results

### Insulin sensitivity measurement and iPSC generation

Individuals in the study have accompanying genome-wide genotyping and gold standard measurement of insulin sensitivity (i.e. steady state plasma glucose-SSPG-derived from an insulin suppression test). Other biometric parameters include age, body mass index, sex and race/ethnicity (**S1 Table**). We generated three to seven iPSC lines from each individual, with no apparent differences in the reprogramming efficiency between IR and IS cells (**S1A Fig**). The complete pipeline for iPSC generation and quality control has been previously described [9]. Briefly, we generated RNA-seq data for 317 iPSC lines from 101 individuals and, after quality control [9], we analyzed RNA-seq data from 310 samples from 100 individuals, of which 48 were IS (149 samples) and 52 were IR (161 samples). The SSPG cut-off to discriminate between IR or IS state was set at 140 mg/dl based on previous publications [15–17] (**S1B Fig**). The average SSPG values were 84 mg/dl for the IS group and 210 mg/dl for the IR group. Finally, samples in both groups were age and body mass index (BMI) matched to avoid possible biases (mean age 57.7 years old and 59.5 in the IS vs. IR group, respectively, and mean BMI 28.5 in the IS vs. 30 in the IR group).

### Differential expression analysis

iPSC lines have been demonstrated to recapitulate many Mendelian diseases, including insulin resistance resulting from severe mutations in the insulin receptor [18,19]. However, the extent to which iPSCs are a valid model for the study of common polygenic forms of IR/T2D is still unknown. To test the hypothesis that iPSCs maintain at least some differential characteristics based on the insulin sensitivity status of the individual they were derived from, we sought to analyze the differential expression (DE) signature between IR and IS iPSC lines. To that end, after alignment and feature counting, RNA-seq counts were normalized and residual expression was computed after adjusting for both technical (sequencing batch, RNA extraction method; modeled as random effects) and biological/population (reprogramming source cell, sex, ethnicity, age, BMI; modeled as fixed effects) covariates (**Figs 1** and **S2)**.

**Fig 2:**
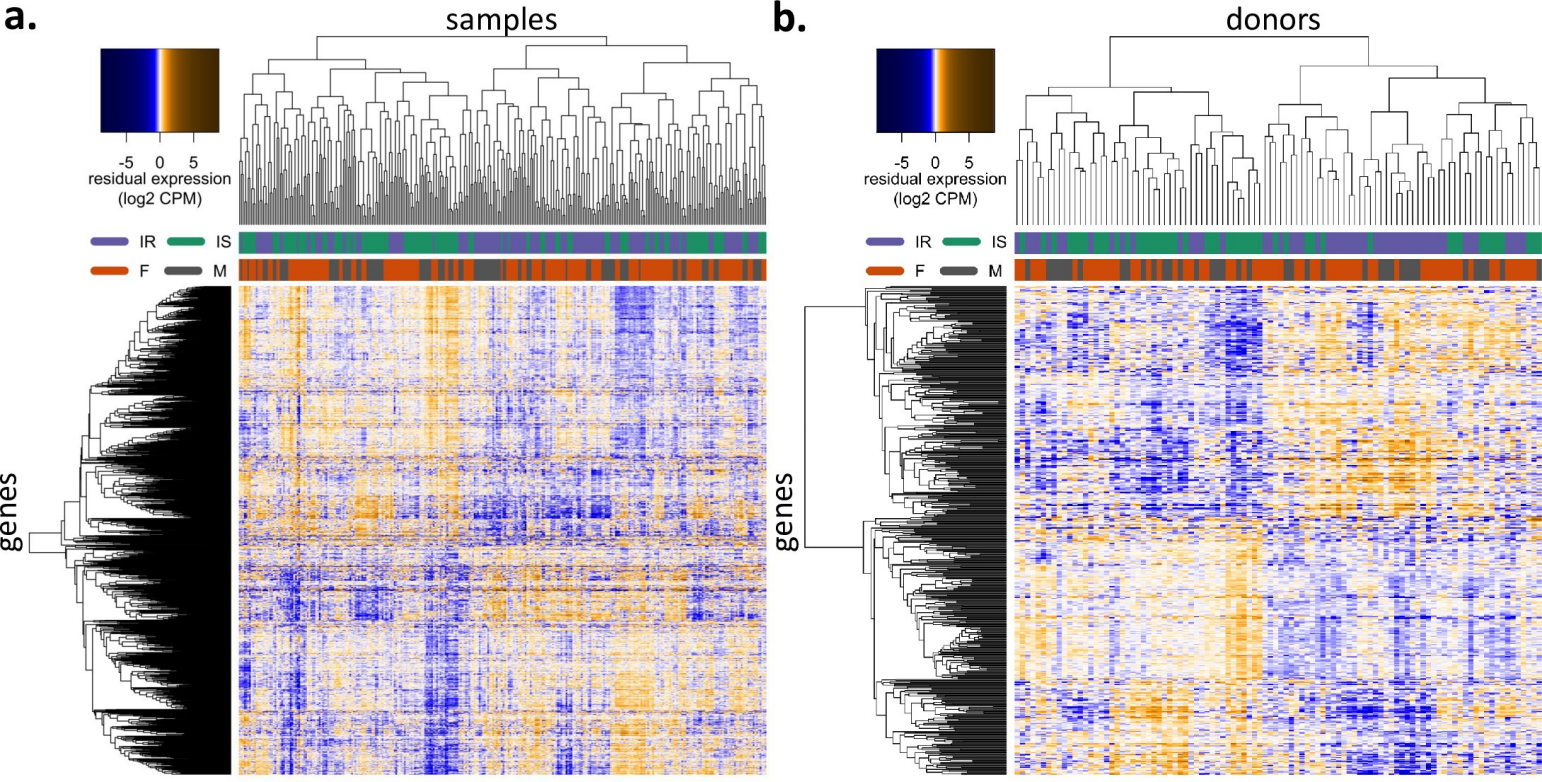
Differential expression analysis. **(a)** All-Samples (AS): 1338 differentially expressed genes with multiple testing corrected p-value<0.05 are shown. **(b)** Average-per-Patient (ApP): top 500 differentially expressed genes identified in ApP are shown. Color scale indicates normalized residual expression levels (blue: low expression, orange: high expression). Columns have been arranged according to clustering of the samples / donors, (purple: insulin resistant, turquoise: insulin sensitive, orange: female, grey: male). Clustering was performed on all samples / donors agnostically of their IR / IS status and sex.

We generated several iPSC lines per individual and thus, the effective sample size of our analyses is the number of patients, not the number of samples. However, since insulin resistance status is an individual-level characteristic, we could not adjust for donor without removing the signal of interest (insulin sensitivity status). Therefore, we analyzed the data in two ways. First, we used all samples (referred to as the AS analysis) without accounting for multiple clones for each individual. Second, we averaged the residual expression levels for all clones of each individual (referred to as the average-per-patient, ApP, analysis). While generalized estimating equations, mixed models or similar techniques can be used to compute residual expression levels taking into account the correlation of samples from the same individuals, these approaches will not allow constructing co-expression and predictive networks for data with multiple clones per individual. We therefore decided to use both the AS and ApP approaches, thereby consistently using the same residual expression levels in all analyses. Importantly, iPSC lines from the same individual cluster together **(S3 Fig)** and samples from IR donors tend to cluster together as do samples from IS donors, whereas this is not the case according to sex **(Fig 2).**

We identified 1,338 differentially expressed genes between IR and IS samples in the AS analysis (FDR adjusted p-value < 0.05) whereas in the ApP analysis no significantly differentially expressed genes were identified (**S2 Table**). However, the comparison of the rank of the test statistics for the 500 most differentially expressed genes from both analyses shows consistent results, with very similar ranks for AS and ApP DE analysis (Spearman correlation coefficient = 0.66, median rank in both AS and ApP = 308, paired Wilcoxon signed-rank test P = 0.90) (**Fig 2**). This result suggests that the lack of statistically significant DE genes in the ApP analysis is due to the reduced sample size. We also performed sensitivity analyses: a) blocking by patient ID, 947 of the top 1338 most differentially expressed genes were shared with AS residuals (71% overlap) and b) using SSPG as a continuous score instead of performing an IR vs IS dichotomization showed that 840 of the top 1338 DE genes with this approach were overlapping with AS residual DE gene list (63%). As our holistic approach to compare IR and IS states was based and scaled to the entire transcriptional network, we used differential expression analysis to leverage our key driver analysis.

### Co-expression network analysis

**Fig 3:**
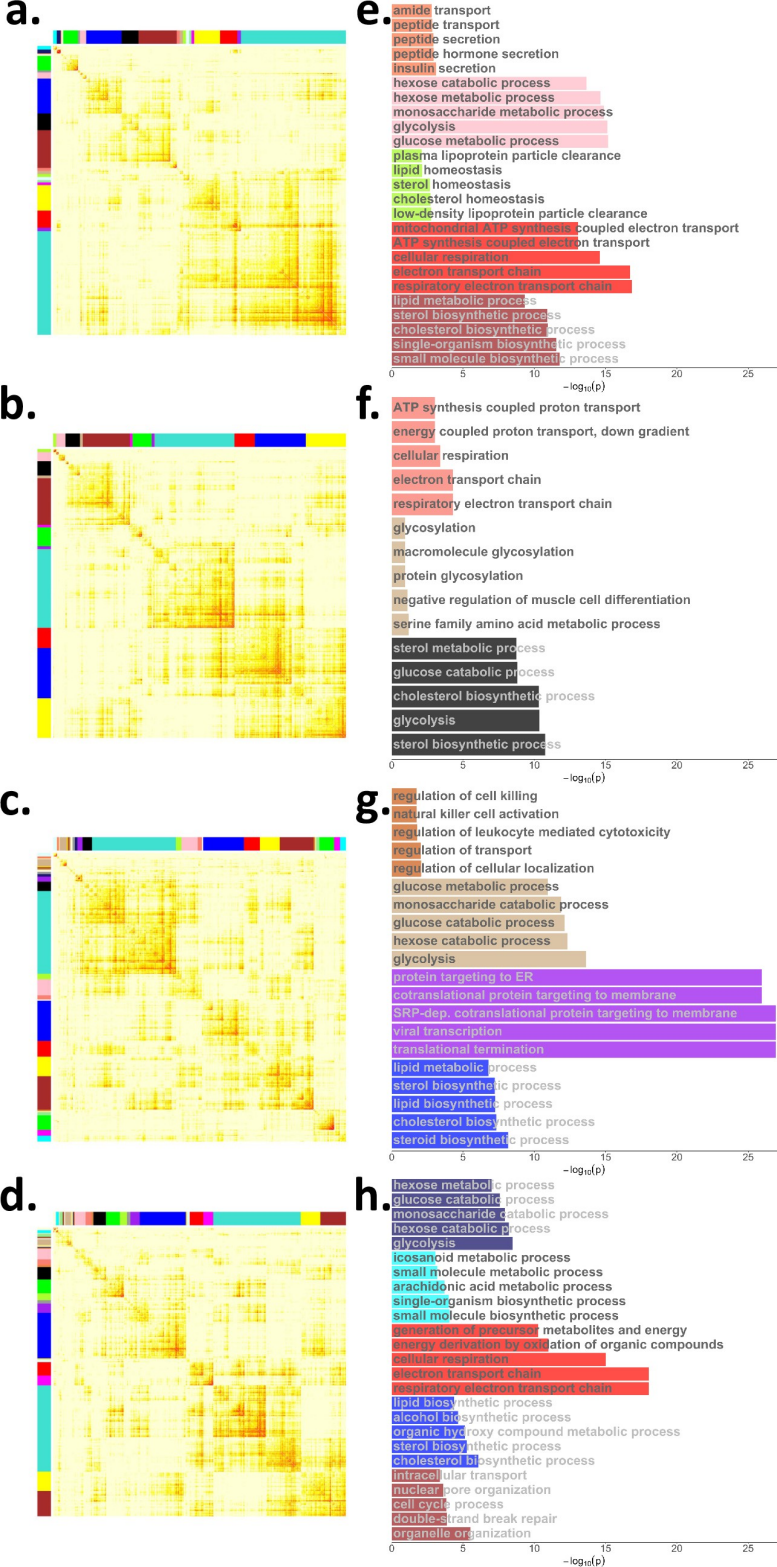
The topological overlap matrix (TOM) indicating co-expression between genes and their corresponding pathway enrichment analysis of the IR and IS co-expression network in AS and ApP. The color bars on top and to the left of each TOM indicate different co-expression modules (WGCNA labels co-expression modules according to a sequence of colours; each colour corresponds to a different module with no further meaning of the colour labels). Only genes included in co-expression modules are shown. The color scales for the different TOMs depend on the soft power parameter used by WGCNA and hence we have not provided color scales for these heatmaps; the main point of the TOM is to show the connections (i.e. co-expression) between blocks (i.e. modules) of genes. Each module of co-expressed genes will correspond to different biological functions and thus an annotation of the modules will allow us to select modules to seed the predictive networks. **(a)** TOM of IR in AS; **(b)** TOM of IS in AS; **(c)** TOM of IR in ApP; **(d)** TOM of IS in ApP. **(e)-(h)** Pathway enrichment analysis results for selected modules from each co-expression networks in (a)-(d).

We trained four co-expression networks (**Fig 3A–3D**) [14,20]: for each of the AS and ApP adjusted expression residuals, we built one network for IR samples and another one for IS samples. Co-expression networks identify groups of genes (modules) with highly correlated expression patterns across samples, indicating that they are involved in similar biological processes. We used gene set enrichment analysis (GSEA) [21,22] and the gene ontology (GO, C5 biological processes, v5.1)[23,24] gene sets from the Molecular Signatures Database (MSigDB) [21] to test the modules of each co-expression network for enrichment in insulin or metabolism related pathways. Specifically, we identified 5 relevant modules in the AS IR network (corresponding to a total of 1,565 genes) -reflecting glucose, lipid and cholesterol metabolic processes and mitochondrial function pathways-, 3 in the AS IS network (430 genes)–glycolysis, cholesterol biosynthesis and electron transport chain-, 4 in the ApP IR network (1,689 genes)–glucose metabolic process, lipid and cholesterol associated processes and regulation of cellular localization-, and 5 in the ApP IS network (2,791 genes)–hexose metabolic process, respiratory electron transport chain, cholesterol, lipid and small molecule biosynthesis-(**Fig 3E–3H**). To evaluate the significance of correlations between gene pairs in the co-expression network modules, we permuted the AS and ApP residuals 10 times and computed the correlation between all pairs of genes in each module per co-expression network (AS IR, AS IS, ApP IR, ApP IS). The correlations from the permuted data formed a background distribution to calculate the p-values of the actual co-expressed gene pairs, which showed that the majority of the pairwise correlations between genes were significant in two or more modules for each of the four networks **(S4 Fig).**

### Predictive networks

Seeding genes for each predictive network were obtained by expanding the set of genes in the selected modules from the corresponding co-expression network by including all genes connected to any of the selected module genes in k = 3 or fewer steps in a prior, cell type-specific network derived from public gene and protein interaction databases: ConsensusPathDB (CPDB) [25] and MetaCore (v6.24 from Thomson Reuters). The above seeding gene selection process ensures that genes impacting insulin sensitivity are included, while reducing the feature space by excluding irrelevant genes to train the predictive network. Our final seeding gene sets consisted of 7,250 (AS IR), 3,797 (AS IS), 8,183 (ApP IR) and 9,712 (ApP IS) genes respectively. This final seeding gene list was used in the top-down and bottom-up predictive network pipeline (see Material and methods section) to build causal network models.

### Key driver analysis (KDA) and ranking

**Fig 4:**
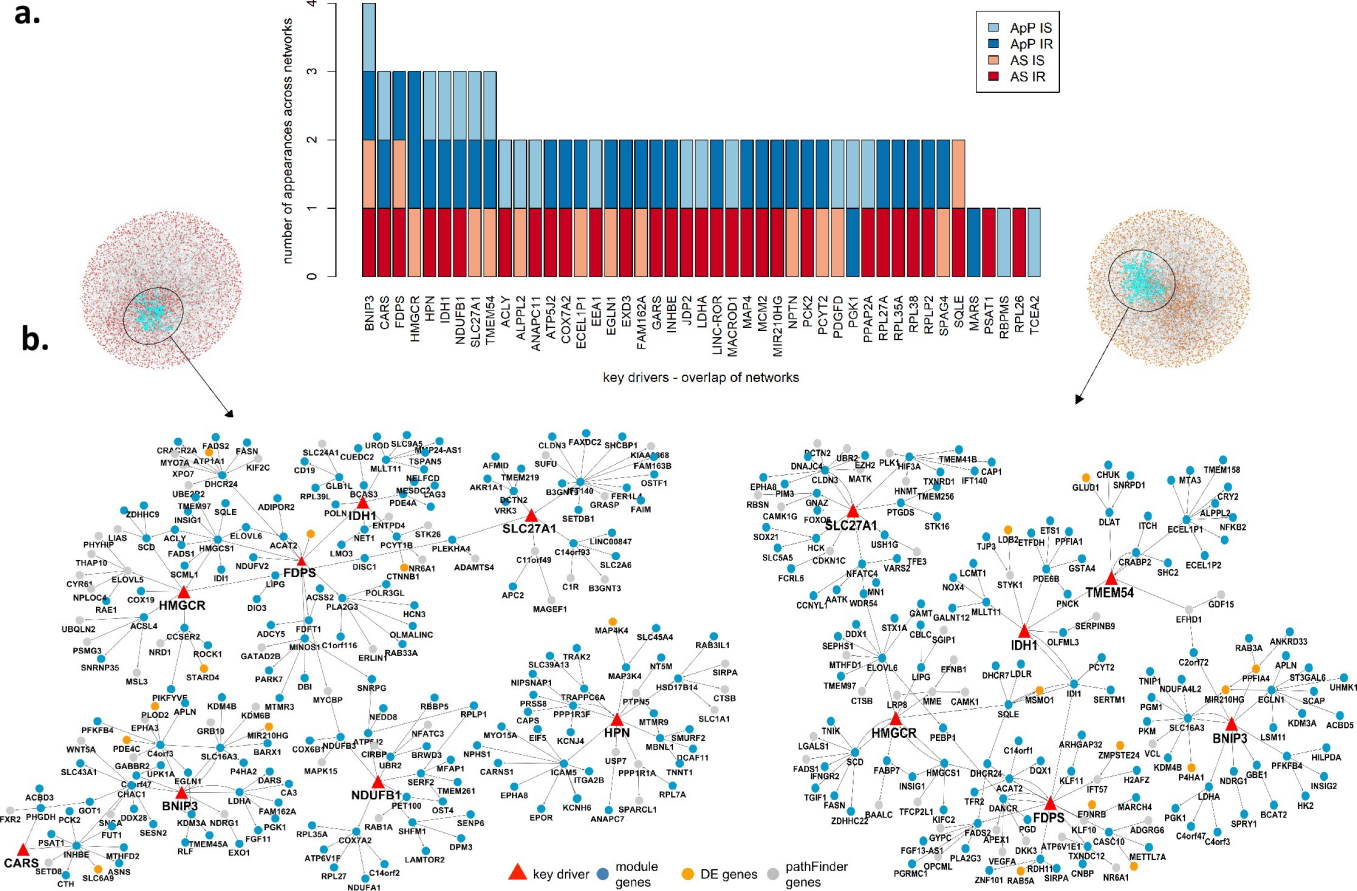
Predictive Causal Network (PCN), key drivers and sub-networks of the tested key drivers. **(a)** key driver replication across networks. For each network a given gene can be identified as a KD based on the co-expression modules and/or the DE genes, and the more often a gene is identified as KD across networks, the stronger the evidence it is implicated in the phenotype; **(b)** sub-networks of top 9 key drivers for IR AS (left) and IS AS (right). We highlight the key drivers (red triangles) and the 2 step downstream genes. Genes from selected modules (blue), DE genes (orange) and expanded genes through pathFinder (grey).

KDA requires a starting set of genes to be analyzed and we ran the KDA multiple times, once for the genes in each co-expression module that was enriched for metabolic pathways, as well as for the DE genes from the AS and ApP analyses (5% FDR for AS and the top 500 DE genes for ApP). Consequently, for each network a given gene can be identified as a KD based on the co-expression modules and/or the DE genes, and the more often a gene is identified as KD across networks, the stronger the evidence it is implicated in the phenotype. Based on this approach, we performed KDA [26] on the four predictive networks and identified, a total of 231 key drivers (KD) in the AS predictive networks and 237 key drivers in the ApP networks. There were 45 key driver genes common to both sets (**S4 Table**). We selected the KDs for further study based on the number of appearances across networks, which rendered 9 genes (*BNIP3*, *CARS*, *IDH1*, *NDUFB1*, *HMGCR*, *HPN*, *FDPS*, *SLC27A1*, *TMEM54*) that are KDs 3 or more times in the AS and ApP networks (**Fig 4A and S4 Table**).

For these top 9 KDs, we further looked at the topology of the local sub-networks around each of them in the four networks and represented the incoming source: selected module genes, DE genes, or expanded genes through pathFinder (**Figs 4B and S5**). The specific module each gene originates from and the enriched functions for those modules can be found in **S6 Fig** and **S3 Table**. In addition, we computed 2 scores (**Table 1**): the sum of the inverse path lengths (i.e. if a gene is k steps down/up-stream of another gene, then the inverse path length is 1/k) from each key driver to the significantly differentially expressed genes in the AS networks and the top 500 most differentially expressed genes in the ApP network (DE proximity score) and second, the difference between the sums of the inverse path lengths from each KD to other KDs downstream of them and the inverse path lengths to other KDs upstream of them (KD dominance score). The higher the DE proximity score, the more there are paths from this KD to DE genes and/or the shorter these paths are; the higher the KD dominance score, the more other KDs are more directly downstream of this KD and/or the fewer other KDs are directly upstream of it.

**Table 1:** Summary statistics and references for the top ranked key drivers. Network appearances: number of appearances across IR and IS networks for AS and ApP DE proximity: sum of the inverse path lengths from each key driver to the significantly differentially expressed genes in the AS networks and the top 500 most differentially expressed genes in the ApP network. KD dominance: the difference between the sums of the inverse path lengths from each KD to other KDs downstream of them and the inverse path lengths to other KDs upstream of them. KD: key driver.

KD	GENE	Network appearances	DE proximity	KD dominance	REFS.
**BNIP3**	BCL2/Adenovirus E1B 19kDa Interacting Protein 3	4	20.30	0.00	[29–31]
**FDPS**	Farnesyl Diphosphate Synthase	3	16.22	1.42	none
**SLC27A1**	Solute Carrier Family 27 Member 1	3	10.85	0.25	[27,28]
**HMGCR**	3-Hydroxy-3-Methylglutaryl-CoA Reductase	3	10.15	0.58	[34,38,44–46,49]
**CARS**	Cysteinyl-TRNA Synthetase	3	7.80	0.00	none
**TMEM54**	Transmembrane Protein 54	3	7.57	1.00	none
**IDH1**	Isocitrate Dehydrogenase (NADP(+)) 1, Cytosolic	3	7.32	-3.25	[85–88]
**HPN**	Hepsin	3	6.67	0.00	none
**NDUFB1**	NADH:Ubiquinone Oxidoreductase Subunit B1	3	6.45	0.00	none

Finally, we performed a directed literature search combining the different KDs with the terms insulin, glucose, and diabetes. The results of the relevant publications associating the KDs to insulin sensitivity are summarized in **Table 1**. Briefly, both *BNIP3* and *SLC27A1* have been strongly associated with insulin resistance phenotypes. Solute Carrier Family 27 Member 1 (*SLC27A1*, also known as *FATP1*) is an insulin-sensitive fatty acid transporter involved in diet-induced obesity and has been associated to IR in skeletal muscle [27,28] and BCL2/Adenovirus E1B 19kDa Interacting Protein 3 (*BNIP3*) is essential for mitochondrial bioenergetics during adipocyte remodeling [29], regulates mitochondrial function and lipid metabolism in the liver [30] and in conjunction with *PPARγ* couples mitochondrial fusion-fission balance to systemic insulin sensitivity [31]. Although informative about the quality of our KDA, the body of publications related to these two KDs decreased the interest on further validation. Among the remaining top KDs, *HMGCR* and *FDPS* have the highest combined DE proximity and KD dominance scores and both genes participate, together with squalene epoxidase (*SQLE*)- another KD that appears in both AS and ApP networks (**S4 Table**)- in the cholesterol biosynthesis pathway. Given, i) the high DE proximity and KD dominance scores, ii) the shared metabolic pathway, iii) the widespread use of statins (*HMGCR* inhibitors) as therapeutic drugs to lower cholesterol levels in patients with high LDL-cholesterol, and iv) the emerging role of *HMGCR* in energy balance, metabolism and diabetes risk [32–35], we sought to validate our KDA, both transcriptionally and functionally, focusing on *HMGCR*, *FDPS* and *SQLE*.

### Network validation

We validated the causal IR/IS networks and the key driver analysis using the DE gene signature from an HMGCR inhibition experiment in iPSC cell lines derived from both IR (n = 6) and IS individuals (n = 6). For each iPSC line in this experiment (**S5 Table**) we generated RNA-seq data in presence or absence of atorvastatin, an HMGCR inhibitor (statin) widely used in patients with hypercholesterolemia [36]. Previous efforts have validated predictive networks through similar approaches [12,37]

The comparison of atorvastatin-treated and untreated samples resulted in a list of 3205 DE genes (**S6 Table**) that showed the highest enrichment for statin action pathway and cholesterol biosynthetic pathway and other related pathways (**S7 Table**), suggesting that HMGCR inhibition triggers a transcriptional compensatory response to balance the decrease in the cholesterol pathway intermediates.

**Fig 5:**
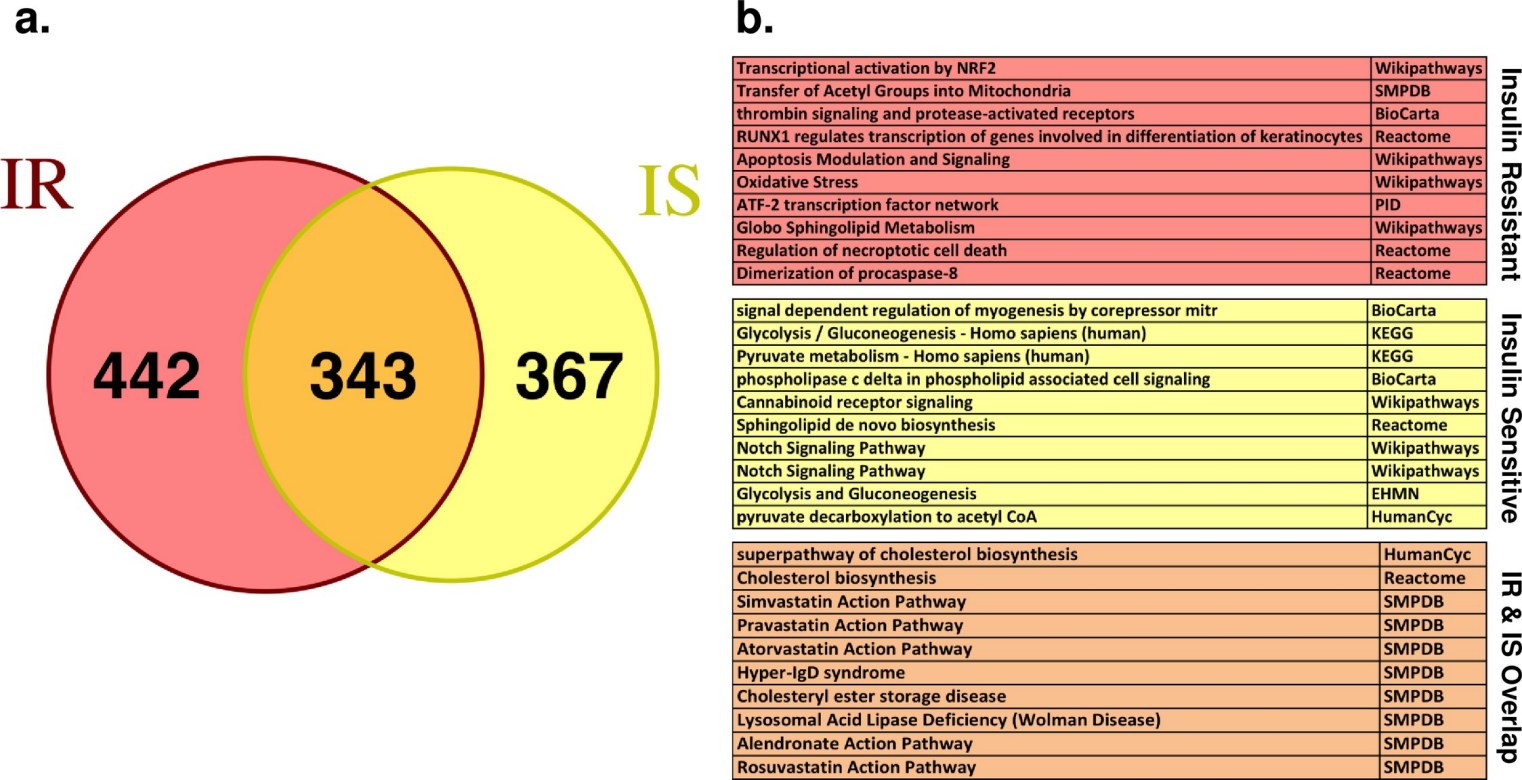
Differential pathway enrichment after HMGCR inhibition in IR vs IS iPSCs. **(a)** Venn diagram for the IR-specific (442 genes), IS-specific (367 genes) and IR/IS overlapping (343 genes) DE genes due to HMGCR inhibition. **(b)** Pathway enrichments for the groups defined in (a). Top-10 significant pathways are shown for each group (full list of pathway enrichment can be found in **S7 Table**).

Next, we wanted to compare the specific responses of IR vs. IS iPSCs to atorvastatin treatment. When considering IR or IS groups independently, the number of DE genes was reduced to 785 (IR) and 711 (IS) (**S6 Table**). Surprisingly the DE genes between IR and IS samples are just partially overlapping (343/785 or 343/711, respectively) (**Fig 5A**) which suggests a differential response to atorvastatin treatment based on the IR/IS status of the donors. Moreover, pathway enrichment analysis (**S7 Table**) for the 442 IR-specific and 367 IS-specific DE genes demonstrated striking differences in the enriched pathways between IR and IS iPSCs (**Fig 5B**), which could be related to the disproportionate incidence of T2D in insulin resistant patients under statin treatment [38]. Although atorvastatin treatment translates the perturbation of a metabolic pathway into measurable transcriptional changes and gives clues about HMGCR functionality and its association to insulin sensitivity, it requires of novel additional analyses to validate the predictive network.

**Fig 6:**
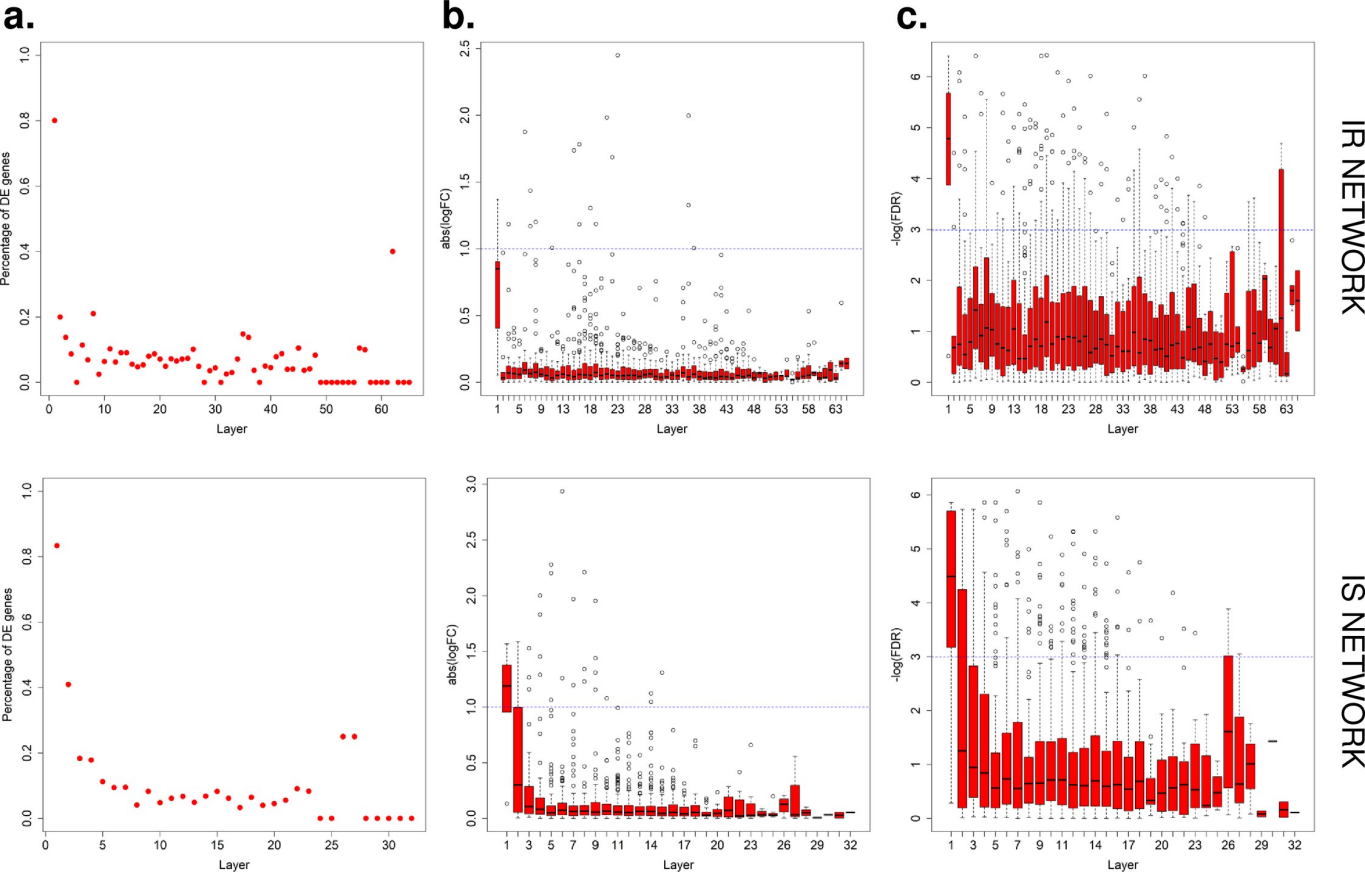
Predictive Network Validation (AS network) by RNA-seq data in Atorvastatin treated iPSC lines. **(a)** The percentage of DE genes decreases as the distance (layers) from HMGCR increases. **(b)** Among the genes located downstream of HMGCR, the fold change decreases as the distance to HMGCR increases. **(c)** The p-value of differential expression analysis from Atorvastatin experiment decreases along the steps down from HMGCR. Upper lane: AS IR network. Lower lane: AS IS network.

To validate our network structure, i) we calculated the percentage of genes in each downstream layer of HMGCR in the network that are significantly altered (FDR<0.05) in gene expression in HMGCR inhibition experiment. We found that for both IR and IS networks more than 80% of the genes in the first layer downstream of HMGCR are DE genes and that this percentage decreases as the distance to HMGCR increases in the network (**Fig 6A**); ii) we also compared DE gene fold-changes (log FC) (**Fig 6B**) and associated significance (-logFDR) (**Fig 6C**) to the AS network topology. Among the genes located downstream of HMGCR in both the IR and IS networks, the fold change and their significance decrease as the distance to HMGCR increases (**Fig 6B and 6C**). The same pattern was observed in the ApP network (**S7 Fig**). Finally, all four predictive networks (AS IR, AS IS, ApP IR and ApP IS) show a significant enrichment downstream of HMGCR for DE genes due to HMGCR inhibition (**S8 Table**).

Thus, our results confirmed that percentage of DE genes, DE gene fold change and associated significance decreases as the distance (number of layer/steps away) from the perturbed target increases, and that DE genes are enriched in the downstream steps of HMGCR compared to non-DE genes. Taken together, the HMGCR-inhibition experiment validated the predictive networks and their topological structure.

### Functional validation

We next sought to functionally validate the prioritized KDs -*HMGCR*, *FDPS* and *SQLE-* in processes associated with insulin sensitivity and in particular, to insulin mediated glucose uptake in relevant metabolic cell types such as adipocytes and SKMCs. To that end, we differentiated the Simpson-Golabi-Behmel syndrome (SGBS) human preadipocyte line [39] and the human SKMC line HMCL-7304 [40] to terminally differentiated adipocytes and myotubes, respectively. We focused our validation efforts on *HMGCR*, *FDPS* and *SQLE* as these three key drivers participate in the same metabolic pathway-cholesterol biosynthesis- and, in addition, statins (*HMGCR* inhibitors] are at the center of an intense debate about their effect on insulin resistance and type II diabetes risk [33,41–45].

**Fig 7:**
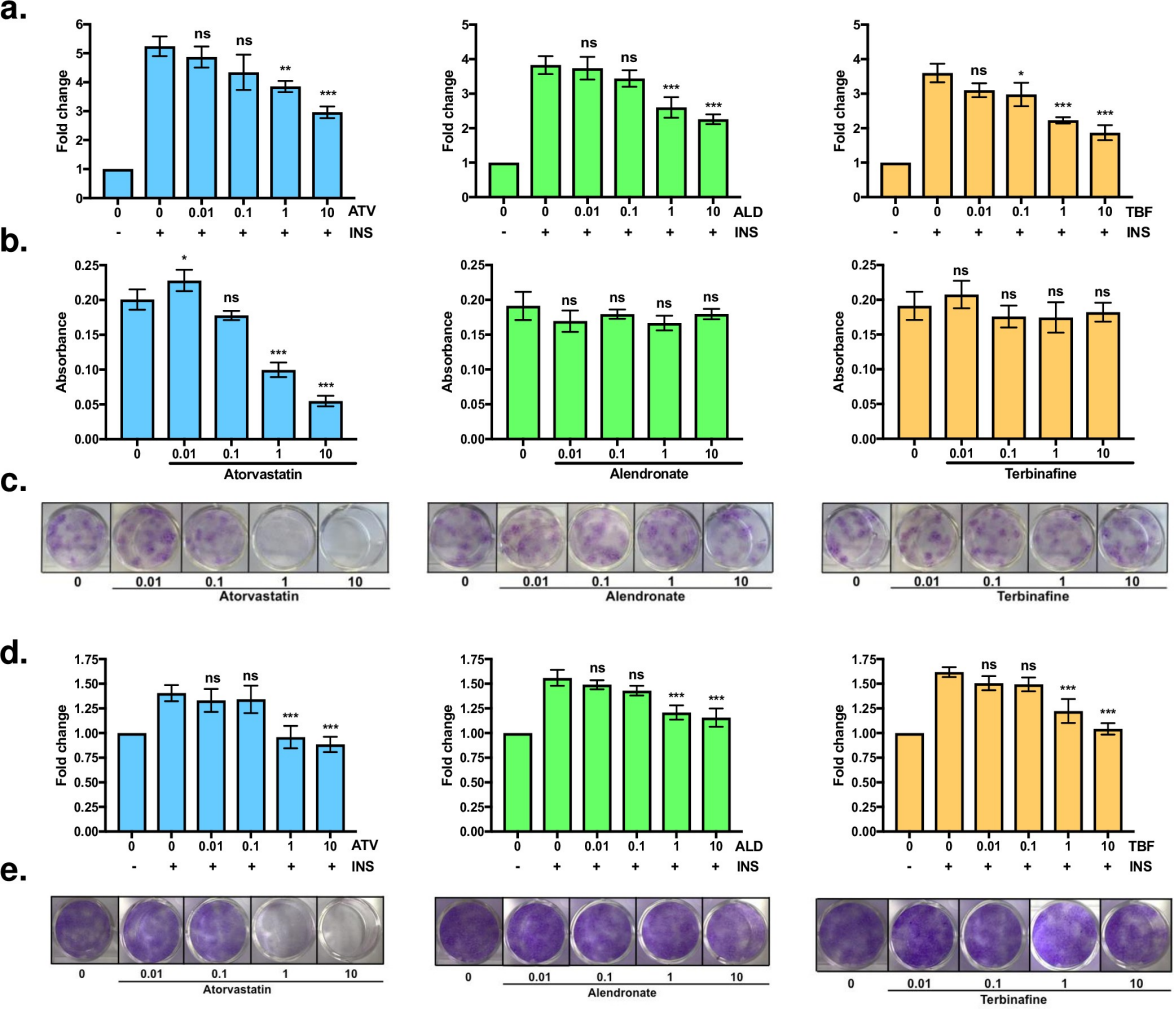
Functional validation of key driver genes. **(a)** Insulin mediated glucose uptake assay in mature human adipocytes. Fold change values are shown with respect to no insulin (-). Each panel shows the effect of varying concentrations of inhibitor for HMGCR (atorvastatin), FDPS (alendronate) and SQLE (terbinafine). **(b)** Adipogenic differentiation assay. The effect of different concentrations of the inhibitors over SGBS adipogenesis is measured by absorbance measurement of Oil-O-Red emission at 500nM. **(c)** Growth assay in human SGBS preadipocytes. Crystal violet staining after 12 days of assay in presence/absence of the inhibitors. **(d)** Insulin mediated glucose uptake assay in mature human SKMCs. See (a). **(e)** Growth assay in human SKMCs. See (b). Results represent mean±SD, and statistical significance was evaluated through One way ANOVA *p<0.05, **p<0.01, ***p<0.001 compared to insulin condition without inhibitors (a and d) or basal differentiation (c).

To functionally inhibit the three candidate genes, we used well-described and widely used chemical inhibitors: atorvastatin (targeting *HMGCR*), alendronate (for *FDPS*) and terbinafine (for *SQLE*) (**S8 Fig**). As shown in **Fig 7A**, all three inhibitors decrease insulin mediated glucose uptake in human adipocytes. However, only HMGCR inhibition demonstrates a detectable decrease of preadipocyte growth and differentiation efficiency (**Fig 7B and 7C)**. Along the same lines, atorvastatin inhibits both insulin mediated glucose uptake and cell proliferation in SKMCs, while alendronate and terbinafine show only a significant effect on glucose uptake (**Fig 7D and 7E)**.

Our results suggest that data driven co-expression and predictive networks combined with key driver analyses are powerful tools for the discovery of novel genes involved in IR.

## Discussion

Although GWAS studies have targeted T2D and insulin resistance- associated glycemic traits, the success in identifying new genes that contribute to insulin resistance risk has been fairly limited. Our group has previously demonstrated that GWAS studies based on gold-standard measurements allows the discovery of novel genes associated with insulin resistance [6] but the power to detect novel loci associated to IR has been limited by sample size. In addition, the genetic complexity and multicellular targets of insulin resistance advocate for the development of new cellular systems and holistic genetic approaches.

With this goal in mind, we generated an iPSC library with accurate measurements of insulin sensitivity that reflects the broad spectrum of insulin responses in human populations. Given our limited sample size (52 IR vs. 48 IS individuals for a total of approximately 300 iPSC lines), and to overcome the limitations of the traditional DE analyses, we developed a more holistic view of the genetic networks associated to IR through the construction of co-expression networks for both IR and IS iPSC lines. In addition, for network construction we performed a dual approach where we used the gene expression values for all samples (AS)(to increase power) or the average per patient (ApP) (to increase stringency). One limitation of our study is that given the complex polygenic nature of insulin sensitivity and given the complexity of the generated data and the multiple sources of variance to gene expression, the multiple corrections that were needed to perform the DE analyses could potentially mask some of the genetic signals associated to insulin sensitivity. Moreover, the extent to which different DE analyses, like using SSPG as a continuous variable instead of dichotomizing IR and IS status, would affect network construction has not been explored in the present study. However, the constructed networks highlighted co-expression modules enriched for cellular functions like respiratory electron transport chain, glycolysis, cholesterol and steroid biosynthesis and glucose metabolism that are intimately associated with insulin sensitivity-associated processes. We performed predictive network and key driver analyses to investigate the central genetic nodes that control the aforementioned modules and functions and thus, are most likely to be involved in the etiology of insulin resistance.

To better delimit and rank our key driver list we considered only the KDs defined as such in both AS and ApP approaches (45 genes) and then we considered the total number of appearances in the 4 constructed networks (AS IR, AS IS, ApP IR and ApP IS), which identified 9 top key drivers. As highlighted in **Table 1,** *IDH1*, *BNIP3* and *SLC27A1 (FATP-1)* have been shown to participate in functions associated with insulin sensitivity or have been directly associated to insulin resistance or type 2 diabetes. Among the rest of the selected key drivers, the top two key drivers with the highest DE path and KD path values, which represents the connectivity of a given KD to DE genes and to other KDs, are Farnesyl Diphosphate Synthase (*FDPS*) and 3-Hydroxy-3-Methylglutaryl-CoA Reductase *(HMGCR*) which coordinately participate in the cholesterol biosynthetic pathway. Moreover, another gene participating in this pathway, *SQLE* is among the 45 KDs shared between both approaches. Meta-analysis of clinical trials with statins (HMGCR inhibitors) have shown an increase in T2D incidence [44–46], which seems to be dose related [35,43] and that affects insulin resistant individuals in a disproportionate way [38]. In addition, alleles in *HMGCR* that lower LDL-C confer an increased risk of developing T2D and individuals with familial hypercholesterolemia are protected against T2D [34,47], leading to speculation that statins affect insulin sensitivity and insulin secretion [45,48], although the exact cellular and molecular mechanisms to such an increase in T2D risk are still not well understood.

The predictive network not only illustrates co-regulated genes in the same pathway but can also demonstrates causality upstream and downstream of a given gene. There have been successful efforts to validate predictive networks [12,37], and therefore, it was critical to show that we could capture the downstream effector genes of key drivers in the predictive networks. Our empirical network validation through HMGCR inhibition demonstrated enrichment for DE genes and log fold change in the downstream proximity of HMGCR, which validates the overall structure of the network. In addition, we observed a differential response to atorvastatin treatment depending the IR or IS state of the iPSC tested, which could be related to the increased incidence of T2D under statin treatment that seems to specially affect IR individuals [34,38,41].

Our functional validation assays in human preadipocyte (SGBS) cells demonstrate that HMGCR inhibition decreases preadipocyte proliferation, and differentiation and insulin mediated glucose uptake in mature adipocytes, similar to previous results in mouse preadipocyte lines [49–51]. In addition, both proliferation and insulin mediated glucose uptake are affected in the human SKMCs (HMCL-7304), treated with atorvastatin. FDPS and SQLE inhibition have also a significant effect decreasing insulin mediated glucose uptake in human adipocytes and myotubes, while not affecting proliferation or differentiation. These results suggest that the effects on proliferation and differentiation of adipocytes and SKMCs are independent of the disturbance in insulin mediated glucose uptake. In addition, *SQLE* inhibition exerts a comparable effect on insulin mediated glucose uptake when compared to *HMGCR* inhibition, which suggests that the underlying mechanisms could be mediated by the deregulation of intracellular or membrane bound cholesterol levels [32,52]. Other proposed mechanisms to affect insulin facilitated glucose uptake include decreased expression levels of *SLC2A4* (*GLUT-4*) and caveolin-1 [51,53], disturbed *RHOA* and *RAB4* signaling lowering *GLUT-4* levels in the plasma membrane [54,55], perturbation of the insulin signaling pathway [55,56] and accumulation of free fatty acids [49,57]. Although our results suggest that the cholesterol biosynthetic pathway is directly involved in the insulin-mediated glucose uptake impairment induced by atorvastatin, it is highly probable that additional mechanisms are participating. In addition, different types of statins could exert differential effects in a concentration, species (mouse *vs*. human) or cell specific context (adipocytes *vs*. skeletal muscle cells). In any case, further studies are necessary to dissect the specific mechanisms by which statins increase the risk of developing T2D.

In summary, our work suggests that: i) iPSC retain a donor-specific signature [9], ii) co-expression and predictive networks combined with key driver analyses uncovered robust candidates to participate in IR, iii) IR iPSCs have a differential response to *HMGCR* inhibition when compared with IS cells and iv) the cholesterol biosynthetic pathway is involved in the insulin-mediated glucose uptake impairment observed in human adipocytes and SKMCs. Taken together, these results suggest that iPSCs technology will offer a novel and sophisticated model for the study of IR and the associated cardiovascular disease, especially when relevant metabolic (adipocytes or SKMCs) and vascular (endothelium) cell types are generated from our iPSC library with accurate measurements of insulin sensitivity.

## Material and methods

### Ethics statement

The study included 201 subjects who had volunteered for prior studies between October 2002 and October 2013 and were in general good health. The individuals were re-contacted and re-consented for this study. Stanford Institutional Review Board approved the study protocol, and all subjects gave written informed consent for study participation.

### Patient recruitment, biological parameters and insulin sensitivity measurement

Patient recruitment and blood sampling were performed as previously described [9]. Insulin sensitivity measurement was perf ormed by a modified insulin suppression test in accordance with Knowles et al.[7] See **S1 Table** for complete demographic data.

### RNA-Seq processing

STAR v2.4.0g1 [58] was used to align RNA-seq reads to the human genome built GRCh37. Using featureCounts v1.4.4 [59], we counted the uniquely mapping reads overlapping genes as annotated by ENSEMBL v70.

### Statistical analyses and data processing

Unless otherwise specified, statistical analyses and data processing steps were done using R v3.0.3 [60]. Code for the various analyses described below has been published on GitHub (https://github.com/gitMarcH/iPSCsInsulinResistance and https://github.com/zhukuixi/KDA).

### Expression data normalization and covariate adjustment

After filtering out lowly expressed genes (at least 1 counts-per-million (CPM) in 30% or more of samples), we have data for 15,294 genes left for analysis. We used the R packages edgeR [61] and limma [62] to normalize the RNA-seq expression data. The edgeR function *calcNormFactors()* is used to compute TMM [63] weights which are applied using the limma function *voom()*. The final normalized expression values that are output by *voom()* are on the log2 counts-per-million (CPM) scale.

All RNA-seq data analyses were performed on expression residuals corrected for the effects of technical (sequencing batches and RNA preparation kits, reprogramming source cell) and patient covariates (sex, ethnicity, age, BMI) in line with existing differential expression analysis literature [64–66]. Batch and RNA preparation kit were adjusted for as random effects using the variancePartition R library [67] whereas reprogramming source cell, and patient characteristics were adjusted for as fixed effects using the limma [60] package. Each of the variables we adjusted for corresponds to technical or biological variation that will mask independent genetic signals of insulin sensitivity. At the same time each of these variables impacts the expression levels of a significant number of genes, and hence, any differences in these factors between the IR/IS groups are likely to confound the biological variation due to IR. The variance partition analysis in **S2 Fig** provides insights into the gene expression variability correlated with these covariates.

As detailed in the results section, due to having multiple clones per patient, we computed two sets of expression residuals: one (AS) using all samples from all clones for every patient and one (ApP) where we averaged residuals per patient.

### Genomic data and expression quantitative trait (eQTL) analysis

We have described these processing and analysis steps previously [9]: “Genotype data were filtered to remove markers with over 5% missing entries, minor allele frequency below 1% and Hardy-Weinberg p value < 10^−6^. Genotypes were phased with SHAPEIT v2.r790 [67], and missing genotypes were imputed with Impute2 v2.3.2 [68] using the reference panel from the 1000 Genomes Project Phase 3 [69]. Markers with high imputation quality (INFO > 0.5;[68] and minor allele frequency over 1% were retained for downstream analyses.

Following standard practice, only individuals of European ancestry were included in the eQTL analysis in order to avoid false positives due to the correlation between ancestry and gene expression. Principal components analysis based on genome-wide genotype data identified 81 individuals of European ancestry for eQTL analysis. eQTL analysis was performed with MatrixEQTL v2.1.1 [70] using the first 5 genotype principal components as covariates. Latent variables were identified in the gene expression data using PEER v1.0 [71]. […]. Cis-eQTL analysis considered markers within 1Mb of the transcription state site of each gene. False discovery rates were computed following Benjamini–Hochberg.” The purpose of the cis-eQTL analysis in this work was to have structural priors for the predictive networks (see “Predictive networks” section below).

### Differential expression analysis

For the expression residuals used for the co-expression and predictive network analyses we adopted 2 analysis streams: one where we use all samples without adjusting for patient ID (AS) and one where we average residuals per patient (ApP). Differentially expressed genes were determined using a linear model, as implemented in the lmFit function from the limma package v3.18.13 in R. Statistical significance was assessed using a cut-off of 0.05 on FDR adjusted p-values.

### Co-expression networks and selection of co-expression modules

We constructed co-expression networks using the coexpp R package [20], which provides an optimized workflow for the WGCNA [14] R package (v1.14–1 used here together with R v3.0.3) for large numbers of genes. The soft threshold powers used for the AS IR, IS and ApP IR, IS co-expression networks were 7.5, 9.0, 7.5 and 9.5 respectively. These were chosen by the pickSoftThreshold() function from the WGCNA package with an R^2^ cut-off of 0.8, unless visual inspection of the R^2^ curve as a function of power revealed a plateau near 0.8 and a lower power achieved an R^2^ of almost 0.8 (e.g. between 0.78 and 0.8). In this case, that lower power was chosen instead. All 15,294 genes that passed the low-expression threshold were used to build the co-expression networks. Seeding genes for the predictive network (specifically, the input to pathFinder) were selected to be the genes in co-expression network modules statistically enriched (FDR < 0.05) for GO terms relevant to insulin resistance related traits (biological processes only).

### Predictive networks

Bayesian networks (BN), which provides a natural framework for capturing causality among highly dissimilar types of data, stochastic processes of biology systems, and noise, have become increasingly popular for modeling biology systems [72–75] due to their inherent capability to integrate multi-scale ‘omics’ data such as genetic context, transcriptomics, proteomics, metabolomics, epigenomics and literature knowledge. While Bayesian networks are useful in deciphering causality of molecular interactions, one fundamental problem is that BN can NOT infer causality among statistically equivalent structures, i.e. multiple network structures with opposite causality direction fit equally well to the data. The problem of partial causality in BN has limited their effectiveness in identifying high-quality key drivers by inducing randomness to the causality of learned structure.

Recently, we developed the top-down and bottom-up predictive network pipeline to build causal predictive network models [76], which leverages the bottom-up belief propagation engine [77,78] as a sub-routine to infer causality among equivalent structures. Biochemical reactions can be modeled as nonlinear perturb-response relations, e.g. hyperbolic response, sigmoidal graded/switched response, rise/fall pulsed response, complex non-monotonic or periodical oscillation [79]. The bottom-up method leverages the nonlinearity of biochemical reactions to infer causality of a molecular interaction, i.e. fitting better to the data along true causal direction than false causal direction, therefore, breaks the statistical equivalence. By integrating the novel bottom-up causality inference approach with (top-down) Bayesian networks, the integrated top-down & bottom-up predictive network platform will result in complete causal network with discerned causality among equivalent structures. This pipeline inherits the advantage of BN in integrating the multi-scale ‘omics-’ data (genetics, genomics, proteomics, metabolomics, epigenomics) to construct multi-scale network models. Consequently, the nodes in a predictive network can represent any variable of interest within the biological system, e.g. levels of gene expression [9,76,80] the genotype of a locus [9], the activity of a protein [76], or the abundance of a metabolite, to name just a few. In addition, the predictive network inherited the capability to predict new molecular phenotype upon genetic perturbations through its internal bottom-up belief propagation engine [77,78]. Also, the genotype data is incorporated as cis-eQTL genes in the model where they are constrained to be the top node (without other parents). As *cis*-eQTLs causally affect the expression levels of neighboring genes, they can serve as a source of systematic perturbation to infer causal relationships among genes [9,81,82]. Consequently, we incorporated *cis*-eQTL genes into each network as structural priors. We used the genes in the selected modules from co-expression networks as seeding genes for predictive network modeling.

To build a predictive network, our platform involves the following steps: i) Evaluate the variance of expression level attributed by every technical and clinical covariate using variance partition to adjust a subset of covariates. ii) Perform differential expression (DE) analysis to derive significant DE genes/proteins/metabolites between groups of interest. iii) Build WGCNA co-expression networks to select a set of modules enriched for significant functions. iv) Extract a list of seeding genes from the selected modules and expand this seeding gene set by prior cell type-specific signaling pathways built separately through pathFinder.

### Key driver analysis (KDA) and prioritization of top hits

The fundamental idea of KDA algorithm is to search for master regulators upstream of a set of user-defined downstream effector genes (in our study, we used the genes from the selected modules and the DE genes) given a causal network structure based on Fisher´s exact test. KDA, was performed using a modified version of the R package KDA [26] that we have published in https://github.com/zhukuixi/KDA. Specifically, first a background sub-network is defined by KDA by looking for a K-step upstream neighborhood round each node in the target gene list in the network. Second, starting from each node in this sub-network, KDA evaluates the enrichment of downstream neighborhoods (for each step size from 1 to K) for the target gene list. K = 6 was used in this paper. This cutoff was chosen based on practical considerations (K will depend on the size and complexity of the network) and while we can use other cut-offs, K = 6 is commonly used in previous studies and is the default value suggested in the KDA software [9,26,83,84]. This K value is preferred because the key drivers within K steps can all be detected. However, as we learned from our knockdown experiment and **Fig 6,** when K gets too large, the impact of a gene on the downstream genes diminishes exponentially. Therefore, we believe that K = 6 is a reasonable choice for this study. Finally, we took the overlap of key drivers identified from the AS and ApP networks and further ranked the top 9 KDs (described in the results section and on **Table 1**).

### pathFinder, a fast graphical algorithm

pathFinder is a graphical algorithm we developed and described in [9]. The purpose of pathFinder is to extract neighborhood structures, specifically to expand an initial set from a larger background network. In particular for this paper we expanded the gene set arising from the selected modules.

### HMGCR inhibition in iPSC lines

iPSCs from 6 IS and 6 IR individuals were used in this validation experiment. These 6 individuals were chosen based on the extremes of the distribution for insulin sensitivity and to reduce the confounding genetic noise that intermediate samples could generate. We selected individuals with an SSPG over 200 for the IR group and individuals with an SSPG below 100 for the IS group). In addition, we selected these samples in order to be able to match gender, age and BMI between IR and IS samples. Full covariate information for these individuals can be found in **S5 Table.** The cells were maintained in feeder-free conditions using mTesr1 (Stem Cell technologies, Inc) supplemented with 1 mM L-Glutamine, 1mM Penicillin-Streptomycin, 0.1 μg/ml Fungizone.) on 5% matrigel coated 6-well TC plates. For passaging, cells were washed once with PBS and treated with pre-warmed 1 mM EDTA (Sigma), incubated at 37 degrees for 1–5 minutes, and resuspended in fresh mTesr1 medium 2 μM Thiazovivin (Millipore). After 12 hours incubation in Thiazovivin, medium was changed daily with fresh mTesr1. Cells were grown to ~90–100% confluency, washed once with PBS and were treated with either DMSO (D) or 1 uM Atorvastatin (A) (Selleckhem) for 12h. Cells were washed once in PBS and harvested for RNA extraction using PureLInk RNA mini kit (Thermo Fisher Scientific). Total RNA was quantified using a Nanodrop (Thermo Scientific). RNA samples with a A260/280 ratio <1.8 or >2.3 were excluded from further processing and the RNA was sequenced using the Illumina HiSeq 2500 system.

### Cell culture

Simpson-Golabi-Behmel syndrome (SGBS) cells (human preadipocytes) were provided by Dr. Martin Wabitsch (Ulm University, Ulm, Germany). SGBS cells were cultured in DMEM/F12 supplemented with 10% FBS, 33uM biotin, and 17uM panthotenate. The SKMC line HMCL-7304 was provided by Institute of Child Health (ICH), University College London. Cells were cultured in SKMC growth medium (PromoCell). iPSCs were generated and cultured as previously described [9].

### Adipogenic and skeletal muscle differentiation

For adipogenic differentiation SGBS cells were grown to confluency and subjected to a two-step differentiation process. Cells were first exposed for 3 days to media composed of DMEM/F12 supplemented with 0.01mg/mL of transferrin, 20uM of insulin, 100nM cortisol, 0.2nM 3,3′,5-Triiodo-L-thyronine, 25nM dexamethasone, 250uM 3-Isobutyl-1-methylaxanthine, and 2uM rosiglitazone. Afterwards, cells were exposed to DMEM/F12 supplemented with 0.01mg/mL of transferrin, 20uM of insulin, 100nM cortisol, and 0.2nM 3,3′,5-Triiodo-L-thyronine for additional 12 days. HMCL-7304 cells were differentiated in presence of SKMC differentiation medium (PromoCell) for 4–5 days before glucose uptake was performed.

For quantification of the effect on adipogenic differentiation, chemical inhibitors for HMGCR (atorvastatin), FDPS (alendronate), and SQLE (terbinafine) were added at day 0 of differentiation at different concentrations (10nM, 100nM, 1uM, 10uM.). Differentiation quantification was performed with Oil Red O. Differentiated SGBS cells were fixed with 10% formalin for 10 minutes at room temperature. After washing with 60% isopropanol, samples were incubated in Oil Red O (Sigma-Aldrich) for 10 minutes at rom temperature. Oil Red O was eluted with 100% isopropanol for 10 minutes. The solution was then transferred into a 96-well plate and absorbance measured at 500nm.

### Glucose uptake

Differentiated SGBS or HMCL-7304 cells cells were pretreated for 24 hours with different concentrations (10nM, 100nM, 1uM, 10uM) of the chemical inhibitors for HMGCR (atorvastatin), FDPS (alendronate), and SQLE (terbinafine). After the preincubation, the cells were starved for 2 hours prior to 30 minute of 100nM insulin stimulation at 37 degrees. Following stimulation, cells were incubated with Krebs-Ringer bicarbonate-HEPES (KRBH) buffer (130mM NaCl, 5mM KCl, 1.3mM CaCl_2_, 1.3 mM MgSO_4_, 25mM HEPES, pH 7.4) containing 100uM 2-deoxy-D-glucose, and 1uCi/ml 2-deoxy-D-[1,2-^3^H]glucose for 10 minutes at room temperature. The cells were then washed with PBS, harvested in 300uL of M-PER lysis buffer (ThermoFisher), and then added to scintillation vials containing 4.75mL of scintillation fluid (Perk Elmer). Radioactive counts were determined with a scintillation counter (Model ID: Beckman LS6500). Excess samples were subjected to BCA assay for protein quantification and normalization of radioactive counts. All samples were represented as fold change compared to the unstimulated (no insulin) condition.

### Growth assay

SGBS or HMCL-7304 cells were plated at 50 or 100 cells/cm2 in 12 well plates and were grown for 12 to 14 days in the absence or presence of 10nM, 100nM, 1uM, 10uM of atorvastatin, terbinafine or alendronate. After the treatment, the cells were fixed in cold methanol for 15 minutes and stained with crystal violet for 10 minutes. Dye excess was washed with water and pictures taken immediately afterwards.

## Supporting information

S1 Fig Reprogramming efficiency and SSPG distribution. (TIF)

S2 Fig Variance partition. (TIF)

S3 Fig Hierarchical clustering of samples. (TIF)

S4 Fig Significance of gene-pair correlation in the four co-expression networks. (TIF)

S5 Fig ApP subnetworks for the top 9 KDs. (TIF)

S6 Fig Module annotation for the genes in the four subnetworks. (TIF)

S7 Fig AS network validation. (TIF)

S8 Fig Cholesterol biosynthetic pathway and location of HMGCR, FDPS and SQLE. (TIF)

S1 Table Patient biometric information. (XLSX)

S2 Table IR vs IS DE genes and rank comparison. (XLS)

S3 Table Go term enrichment for co-expression modules. (XLSX)

S4 Table Key driver analysis. (XLSX)

S5 Table Patient information atorvastatin experiment. (XLS)

S6 Table ATV experiment DE genes. (XLSX)

S7 Table Pathway enrichment atorvastatin experiment. (XLS)

S8 Table HMGCR inhibition DE genes enrichment in downstream of HMGCR in predictive networks. (XLSX)
