# Supplementary figures and images for: Predictive network modeling in human induced pluripotent stem cells identifies key driver genes for insulin responsiveness
Source: PLoS Comput Biol. 2020 Dec 23;16(12):e1008491. doi: 10.1371/journal.pcbi.1008491 (PMC7790417; doi:10.1371/journal.pcbi.1008491)

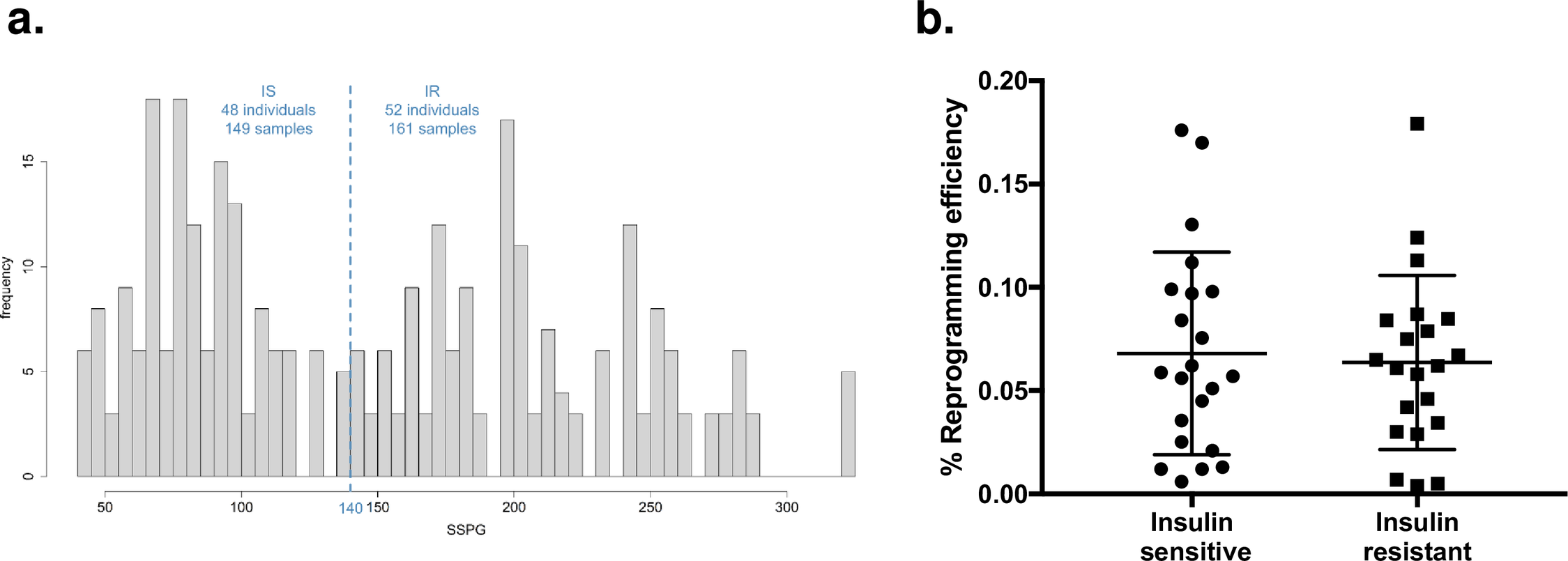

Supplement: S1 Fig — (TIF) [file pcbi.1008491.s001.tif]

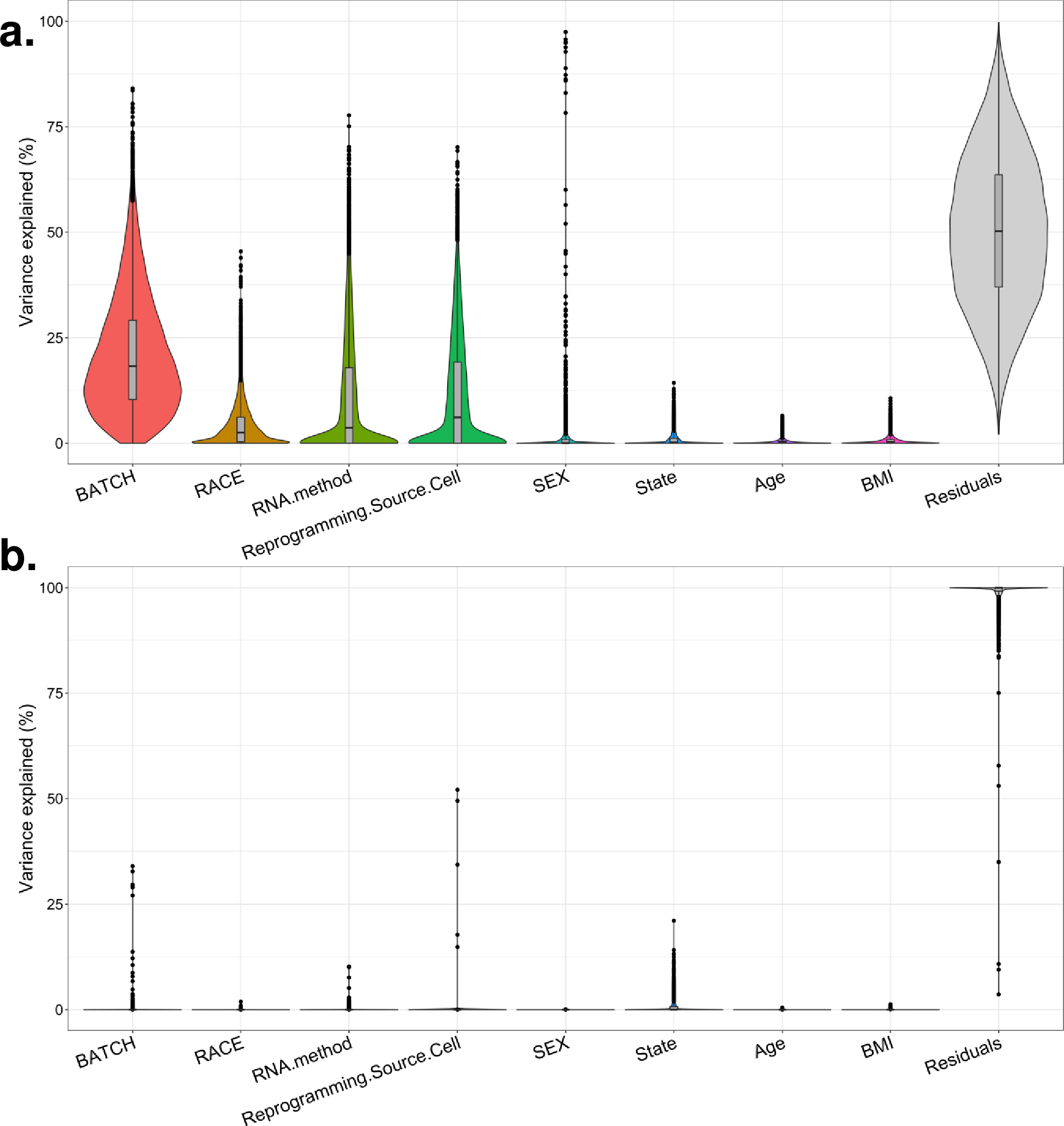

Supplement: S2 Fig — (TIF) [file pcbi.1008491.s002.tif]

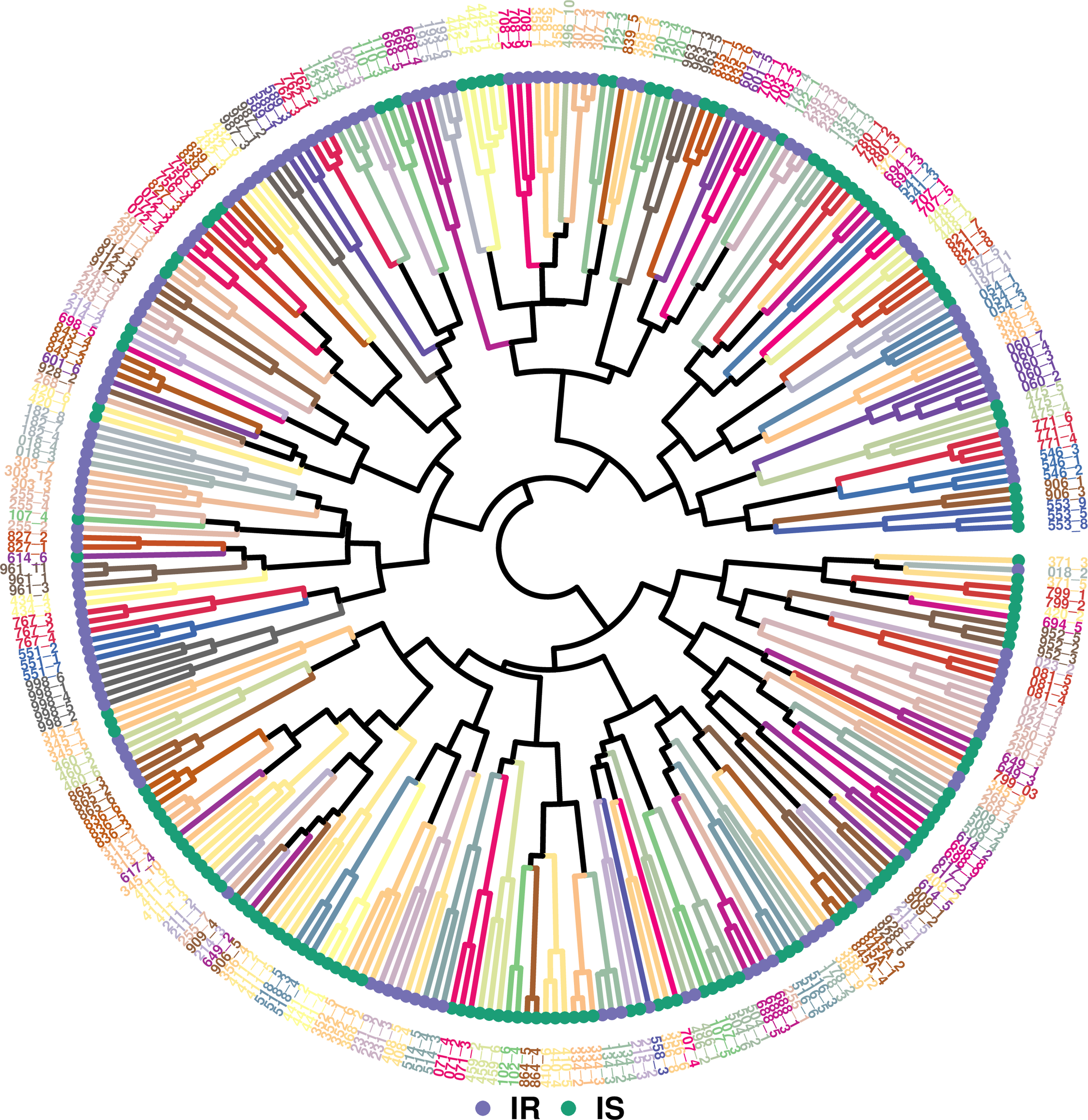

Supplement: S3 Fig — (TIF) [file pcbi.1008491.s003.tif]

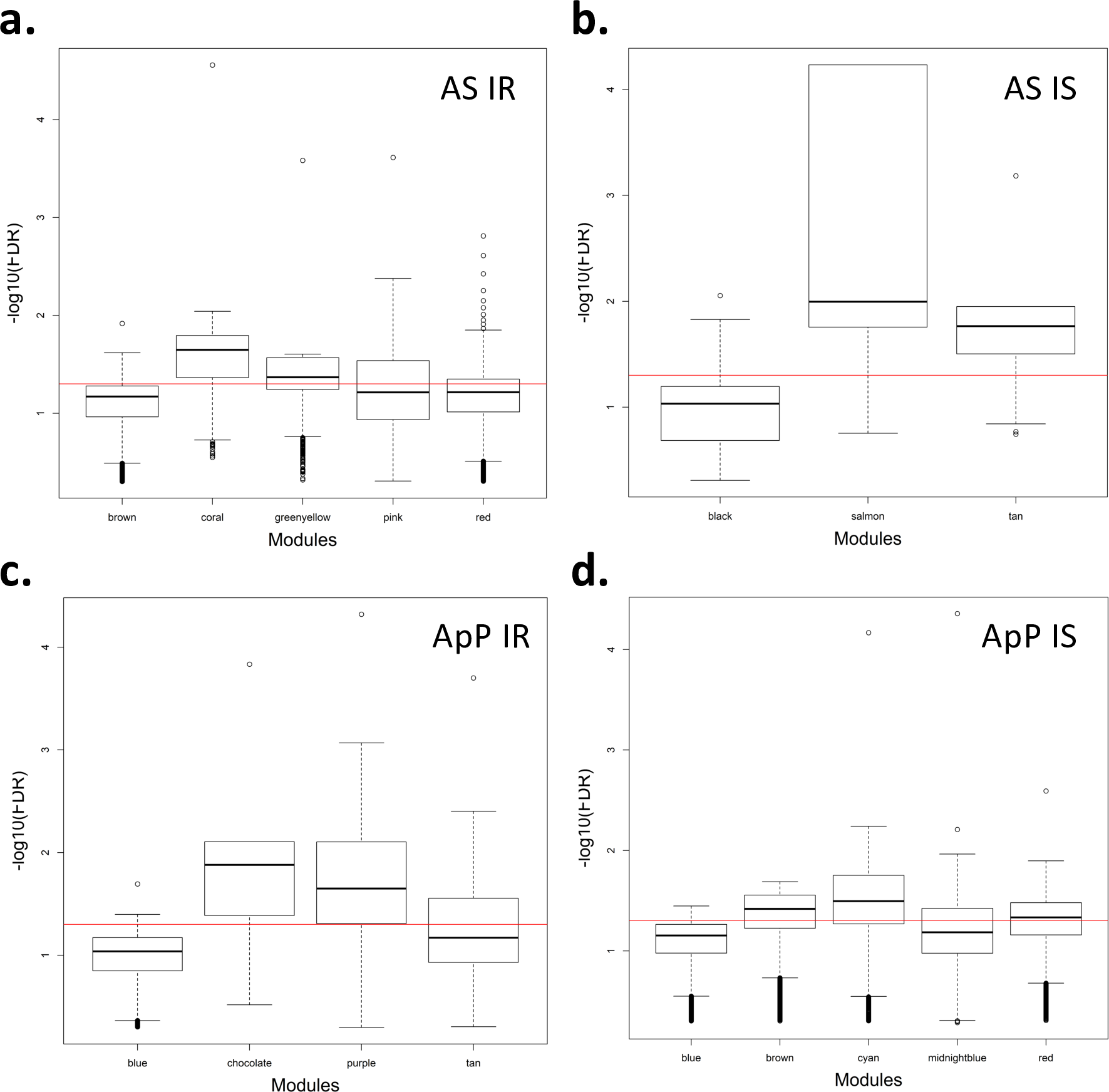

Supplement: S4 Fig — (TIF) [file pcbi.1008491.s004.tif]

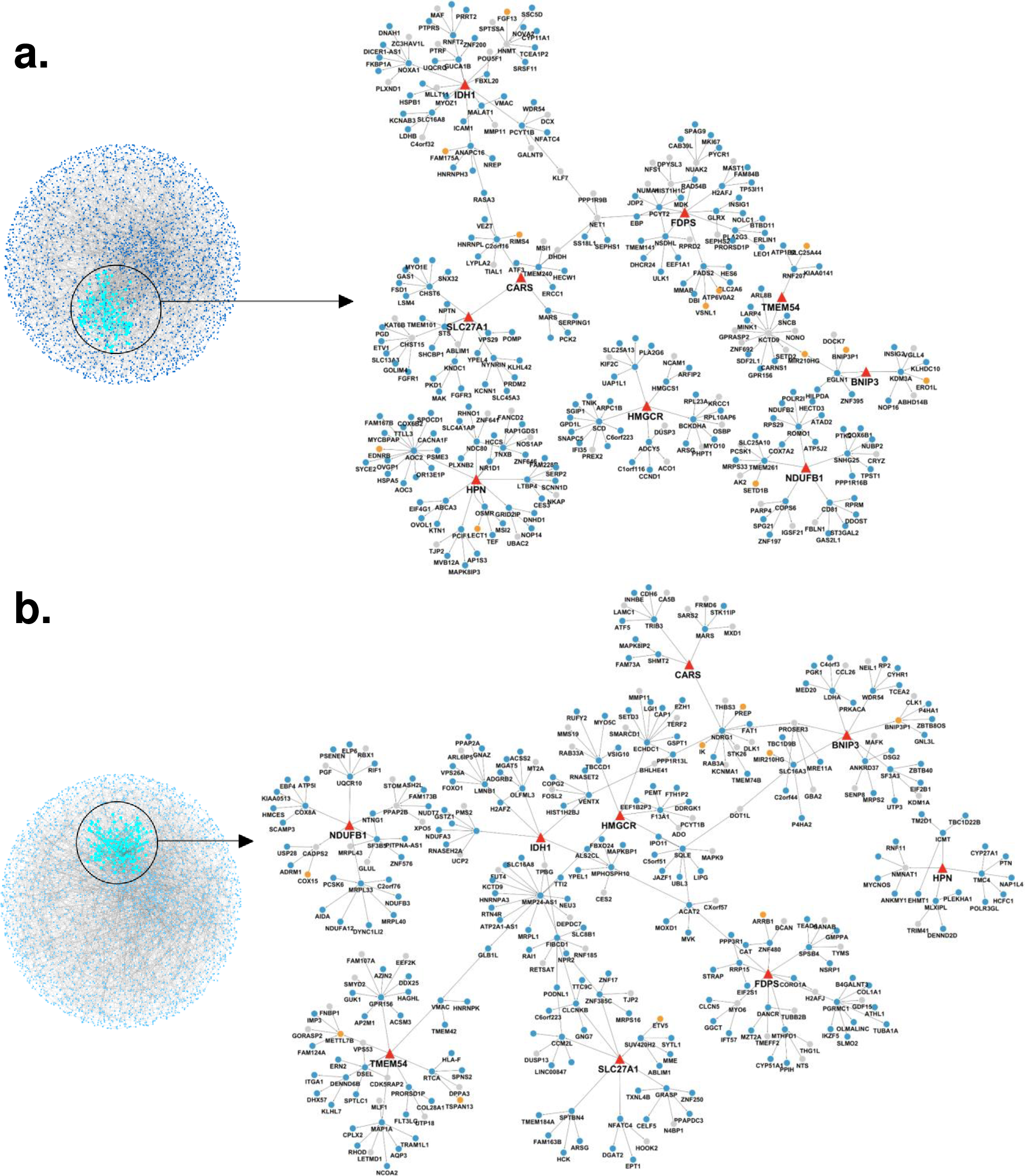

Supplement: S5 Fig — (TIF) [file pcbi.1008491.s005.tif]

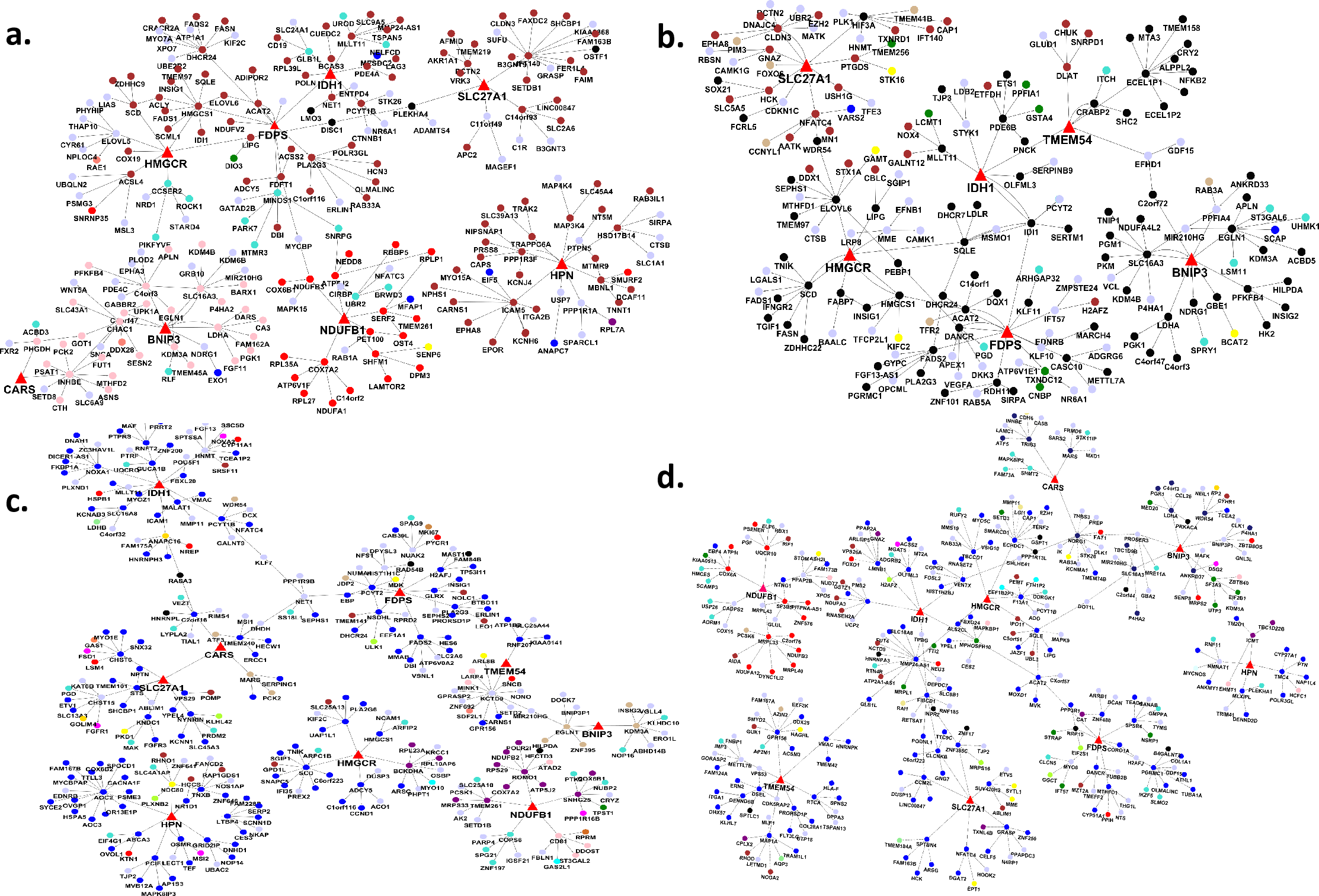

Supplement: S6 Fig — (TIF) [file pcbi.1008491.s006.tif]

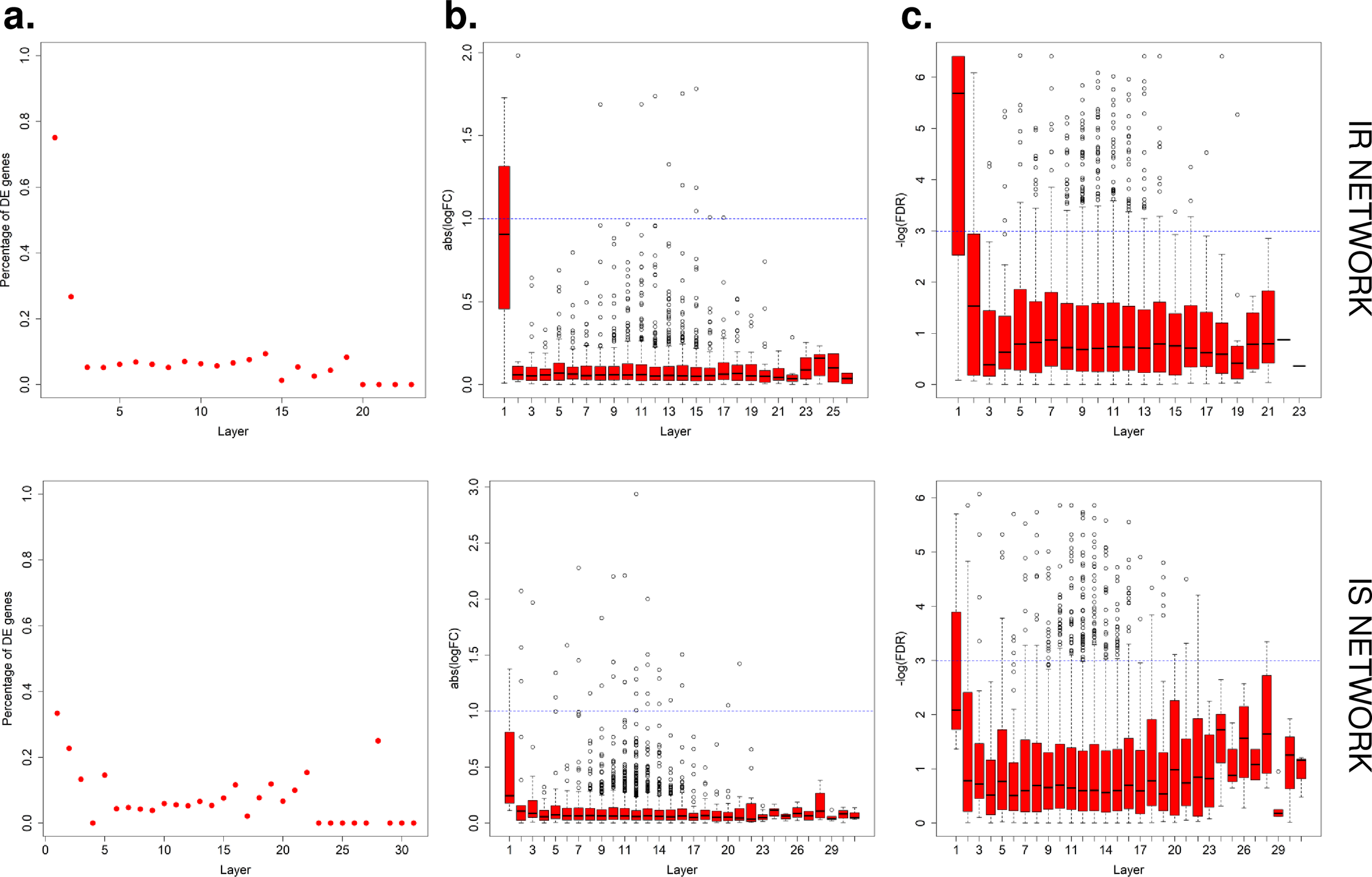

Supplement: S7 Fig — (TIF) [file pcbi.1008491.s007.tif]

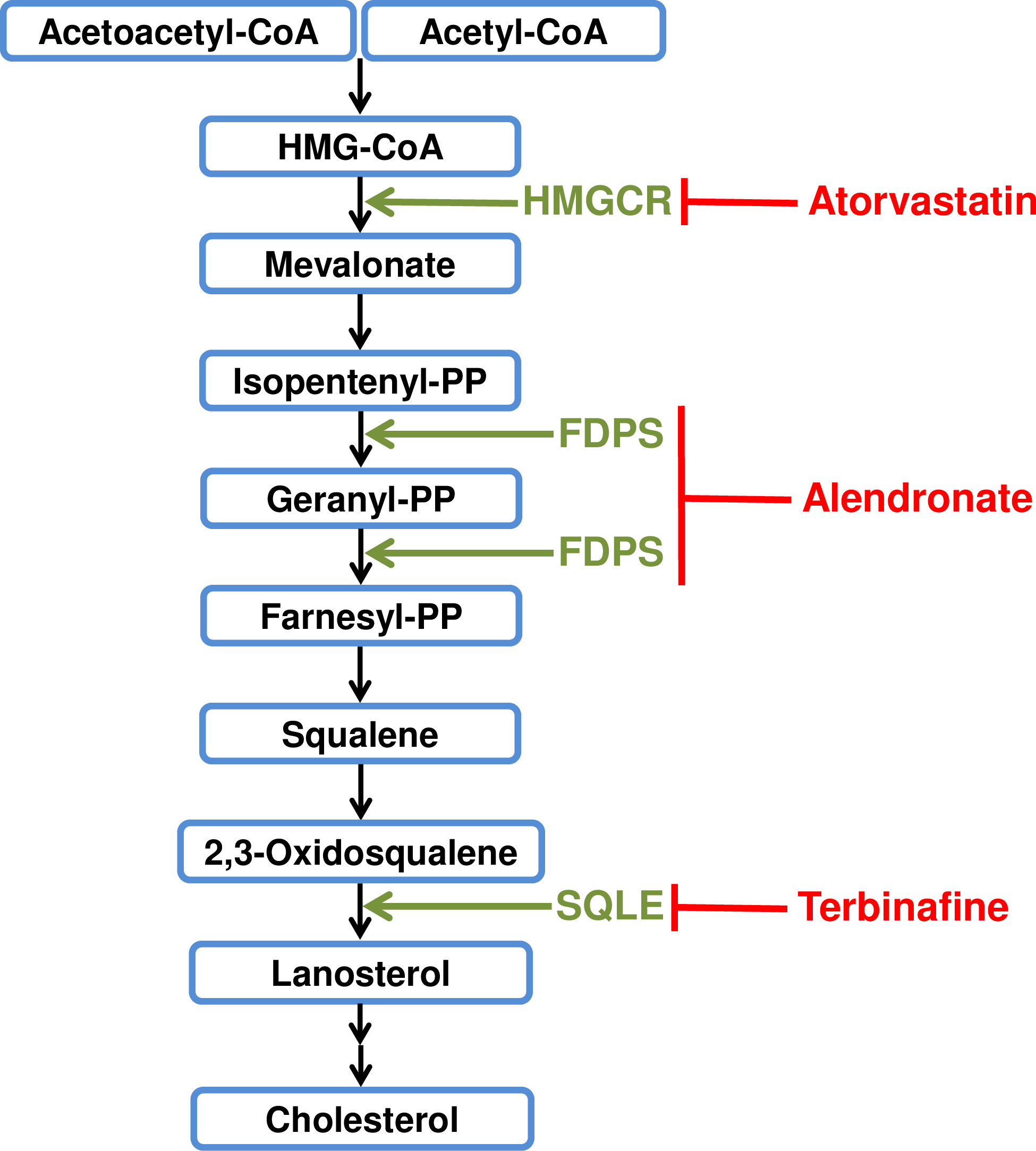

Supplement: S8 Fig — (TIF) [file pcbi.1008491.s008.tif]
